# Rich in Phenolics—Strong Antioxidant Fruit? Comparative Study of 25 Strawberry Cultivars

**DOI:** 10.3390/plants11243566

**Published:** 2022-12-17

**Authors:** Dragica M. Milosavljević, Vuk M. Maksimović, Jasminka M. Milivojević, Đura J. Nakarada, Miloš D. Mojović, Jelena J. Dragišić Maksimović

**Affiliations:** 1 Department of Life Sciences, Institute for Multidisciplinary Research, University of Belgrade, Kneza Višeslava 1, 11030 Belgrade, Serbia; 2 Faculty of Agriculture, University of Belgrade, Nemanjina 6, 11080 Belgrade, Serbia; 3 Faculty of Physical Chemistry, University of Belgrade, Studentski trg 12-16, 11000 Belgrade, Serbia

**Keywords:** electron paramagnetic resonance (EPR) spectroscopy, HPLC-MS, anthocyanins, flavonols, flavan-3-ols, hydroxycinnamic acids, peroxidase, antioxidant, strawberry fruit

## Abstract

Phenolic compounds of 25 newly introduced strawberry cultivars were profiled using spectrophotometry, electron paramagnetic resonance (EPR) spectroscopy, and high-performance liquid chromatography-mass spectrometry. Total phenolic and anthocyanin content (TPC and TACY, respectively), as well as vitamin C, and concentrations of individual phenolic compounds in fruits were evaluated to identify the most promising cultivars according to their phenolic profile. The highest values of TPC, TACY, and vitamin C were recorded in ‘Premy’ (1.53 mg eq GA g^−1^ FW), ‘Sandra’ (30.60 mg eq Pg-3-g 100 g^−1^ FW), and ‘Laetitia’ (56.32 mg 100 g^−1^ FW), respectively. The DPPH and •OH radicals scavenging activity of fruit methanolic extracts was estimated using EPR spectroscopy. All cultivars are almost uniformly effective in the scavenging of •OH radical, while ‘Tea’, ‘Premy’, and ‘Joly’ were marked as highly potent cultivars (over 70%) in terms of DPPH-antiradical activity. Specific peroxidase activities were the highest in ‘Garda’, ‘Federica’, and ‘Rumba’ (0.11, 0.08, and 0.06 U mg^−1^ prot, respectively). ‘Laetitia’, ‘Joly’, ‘Arianna’, ‘Tea’, and ‘Mila’ cultivars were distinguished from others as the richest concerning almost all flavonoids and phenolic acids, including some other parameters of bioactivity. These cultivars could be recommended to consumers as functional fruit foods.

## 1. Introduction

Today, consumer awareness of the impact of fruit intake on overall health and well-being is constantly on the rise. Therefore, the consumption of berry fruits, especially strawberries, has been promoted as valuable due to their overall health benefit properties [1].

Strawberry (*Fragaria × ananassa* Duch.) is a good source of essential vitamins and minerals [2] and significant levels of biologically active components, such as phenolic compounds [3], which, apart from defining consumer acceptance, have essential positive effects on the human diet and health [1]. Strawberry fruit contains natural antioxidant substances such as anthocyanins, flavonoids, and phenolic acids and also has high a level of antioxidant enzymes and oxygen radical scavenging activities, which could effectively and synergistically perform as free radical inhibitors and provide protection against oxidative damage [4]. The diversity and richness in the content of phenolic compounds make strawberries recommendable for human consumption. They can help in reducing incidences of chronic diseases, elevated blood pressure, and platelet aggregation, while at the same time having a positive effect on the immune system, by means of good anti-inflammatory, antibacterial, and antiviral response [5]. Considering that strawberries represent 40% of the total berry consumption worldwide [6], the health-promoting potential of strawberries implies their serious contribution as a dietary source in the human diet.

The phenolic content of strawberry fruits can vary depending on the cultivar, growing location, maturity stage, agricultural practice, and environmental and storage conditions [7,8,9]. In many studies, the antioxidant properties of strawberries are mainly linked to the polyphenols they are rich in [10,11,12,13]. A significant part of the antioxidant capacity of these fruits comes from flavonoids that are subdivided into several subgroups: flavones, flavonols, flavanones, flavanonols, flavanols or catechins, anthocyanins, and chalcones [14]. Anthocyanins are quantitatively the most represented in strawberry, responsible for the red color of the fruit, but they also have strong antioxidant properties [15]. The predominant anthocyanin in strawberry fruit is pelargonidin-3-*O*-glucoside [16,17,18]. The main biological functions of flavonols in fruit are attributed to protection from harmful UV light and pathogens [19]. In addition to their involvement in biochemical signaling pathways, their antioxidant effect is extremely important for a large number of physiological and pathological processes. Flavan-3-ols are usually denoted by the term ‘flavanols’, representing a subgroup of flavonoids that are powerful antioxidants mainly abundant in the external tissues of fruit [20]. Major flavonoids in strawberries are catechin (belonging to the flavan-3-ol group), as well as quercetin and kaempferol derivatives (i.e., flavonols group) [18,21], which have been shown to have antioxidant and anticancer properties [10,22,23]. Moreover, hydroxycinnamic acid derivatives play an important role in browning reactions during maturation due to their abundance and diversity in fruit flesh [24], represented mainly by coumaroyl glucose in all cultivars.

Hakala et al. measured high concentrations of ascorbic acid, potassium, magnesium, iron, zinc, and calcium in strawberries [21]. Ascorbic acid has many roles, but one of the most important is the capacity to act as a reducing agent, increasing the effects of oxidase enzymes by reducing *o*-quinones to *o*-diphenolics [22]. This indicates that ascorbic acid also acts as an antioxidant by preventing DNA damage caused by free radicals, quenching oxidants that can lead to the development of cataracts and endothelial cell dysfunction, and that it can protect against atherosclerosis by reducing leukocyte adhesion caused by low-density lipoproteins [23].

In protection against oxidative damage, various oxidase enzymes can also play a significant role. Their increased activity in fresh strawberries can cause significant quality deterioration, including loss of color and texture and the formation of unwanted brown pigments [25]. Peroxidases (POD; EC 1.11.1.7) catalyze the oxidation of structurally diverse phenolic substrates using reactive oxygen species over three basic cycles: (1) in the peroxidative cycle, as a result of oxidation, a phenoxyl radical is formed; (2) in the oxidative cycle they produce H_2_O_2_; and (3) in the hydroxyl cycle, a highly reactive hydroxyl radical (•OH) is formed [26]. Together with polyphenol oxidase (PPO; EC 1.14.18.1), they are responsible for the ‘enzymatic tanning’ of fruits, which leads to the rapid degradation of nutritional and structural components and drastically shortens the shelf life and acceptability by consumers [27]. 

Our research aim was to quantify and characterize phenolic compounds in 25 strawberry cultivars and correlate them with their antioxidant capacity. Thus, phenolic components may be used as a selection criterion for defining the quality of strawberry fruit with well-demonstrated potential health benefits. The intercultivar variation of phenolic profiles could be employed for the promotion of promising cultivars with improved quality traits that fulfill consumer preferences, contributing to the ‘educated consumer concept’.

## 2. Results and Discussion

### 2.1. Phytochemical Composition

The concentration and composition of phenolic compounds strongly affect the sensory-organoleptic properties and nutritional values of strawberry fruit, thus contributing to their possible health benefits. Therefore, the increase in consumption of high-quality strawberry fruit should be associated with both high consumer acceptance, due to its sensory attributes, and the presence of bioactive compounds.

Previous studies reported variation in phenolic content among strawberry cultivars focusing on polyphenol-rich cultivars that may thus be an important source of health-promoting compounds in the human diet [3,18,28]. Besides phenolic richness, content of vitamin C has been reported to be an effective enhancer of the oxidative homeostasis of strawberry fruit [28,29], so cultivars rich in these functional ingredients should be promoted as promising.

The results of the total phenolic content (TPC) of 25 strawberry cultivars are given in Table 1. 

TPC of strawberry extracts varied from 0.46 to 1.53 mg eq GA g^−1^ FW. Cultivars ‘Premy’, ‘Aprika’, ‘Rumba’, ‘Vivaldi’, and ‘Sibilla’ were dominant in TPC (1.53, 1.41, 1.40, 1.37, and 1.33 mg eq GA g^−1^ FW, respectively), while ‘Irma’, ‘Alba’, ‘Roxana’, and ‘Jeny’ had the lowest TPC (0.46, 0.49, 0.49, and 0.50 mg eq GA g^−1^ FW, respectively). A previous study [30] showed that strawberry fruits were a good source of TPC, with reported values standing in line with our results.

Strawberry fruits are characterized by the high content and moderate diversity of anthocyanins [31]. In the present study, total anthocyanin content (TACY) was superior in ‘Sandra’ cultivar with a value of 30.60 mg eq Pg-3-g 100 g^−1^ FW, followed by ‘Arianna’ and ‘Vivaldi’ (26.05 and 24.92 mg eq Pg-3-g 100 g^−1^ FW, respectively). These results are comparable to the content of anthocyanins determined by other authors in various strawberry cultivars [32,33]. In contrast, an extremely low TACY value was recorded in ‘Jeny’ (3.08 mg eq Pg-3-g 100 g^−1^ FW). Pelayo et al. [34] reported that the distribution of anthocyanin pigments in the fruit tissue of different strawberry cultivars is not uniform, which is also reflected in differences in the visual external color of the fruit.

A comparison of homogeneous subsets of tested strawberry cultivars demonstrated that the maximum value of vitamin C content was recorded in ‘Laetitia’ (56.32 mg 100 g^−1^ FW), while ‘Sibilla’ reached its lowest point (32.27 mg 100 g^−1^ FW). Previously published average values of vitamin C in strawberry fruits [30,35] are much lower than those in our study, which indicates that the majority of tested newly introduced cultivars are an excellent source of vitamin C. Taking into account its antioxidant properties and metabolic functions [36], vitamin C is an important indicator of internal fruit quality, along with phenolic compounds.

Within a Fragaria species, the overall content and changes of total phenolics, anthocyanins, and vitamin C are strongly affected by cultivar, more pronounced than the effect of environmental conditions [37]. Therefore, cultivars rich in these components can be recommended as valuable sources of health-related compounds for fresh consumption.

### 2.2. Antiradical Activity

Reactive oxygen species (ROS) are produced during the normal growth and metabolism of plants [38]. At low concentrations, ROS are key regulators of many physiological processes in plants such as growth and development, cell cycle, programmed cell death, hormone signaling, and biotic and abiotic stress responses [39,40,41]. At higher concentrations, ROS can evince detrimental effects by damaging membranes, proteins, chlorophyll, and nucleic acids [42,43]. Hence, plants have developed different defense mechanisms, based on antioxidative metabolic compounds and enzymes, to convert ROS into less harmful products, thus regulating ROS homeostasis. Due to this feature, there is a constantly increasing interest in the investigation of naturally occurring antioxidants from plant material, where the fruits can be regarded as a ‘gold mine’.

It was observed that anthocyanins have ROS-scavenging capacities up to four times stronger than analogs of vitamin E and C, due to their high reactivity as proton and electron donors, ability to stabilize and delocalize unpaired electrons, and capacity to chelate transition metal ions [44]. In this regard, electron paramagnetic resonance (EPR) spectroscopy has been successfully applied to determinate radical scavenging activity in different foodstuffs rich in phenolic compounds [44,45,46]. EPR spin-trapping is a highly specific method that enables distinguishing between different short-lived free radical species, each obtaining characteristic EPR spectra of the corresponding spin-adducts [47]. Spin-trap DEPMPO (5-(diethoxyphosphoryl)-5-methyl-1-pyrroline-N-oxide) attracted a lot of attention because of its high sensitivity, adduct stability, and the ability to differentiate between various trapped radical species [48]. Although it is an excellent method for obtaining the specific radical fingerprint, only a few papers on the measurement of free radical scavenging activities of anthocyanin-enriched strawberry fruits by EPR have been published [35,49].

As shown in Table 2, strawberry extracts induced a decrease in the EPR signal intensity of DEPMPO/OH spin-adducts due to competition for the hydroxyl radical between the spin-trapping agent and the antioxidants present in the extract. All tested strawberry cultivars demonstrated high •OH radical scavenging activities (93–96%).

**Figure 1:**
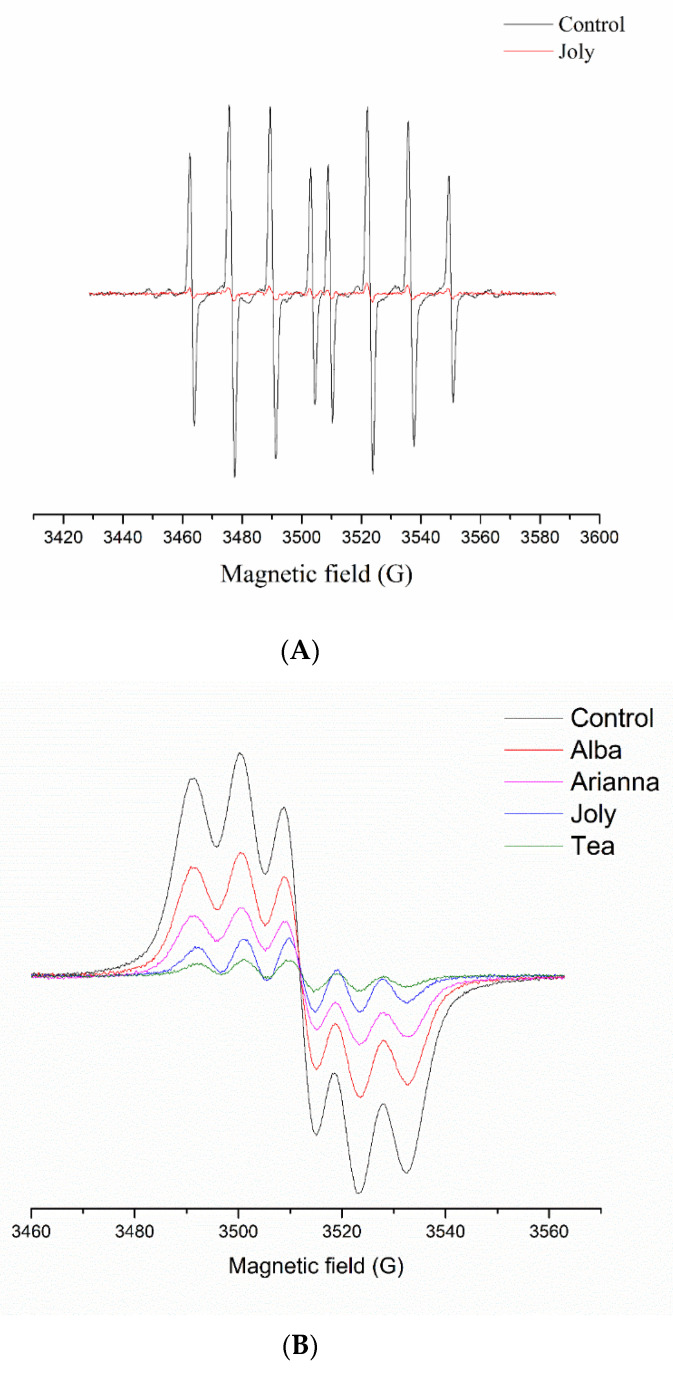
A representative spin-trapping electron paramagnetic resonance (EPR) spectra. EPR spectra of DEPMPO/OH adducts generated by an in vitro Fenton reaction (**A**) and set of individual EPR spectra of DPPH free radicals (**B**).

In Figure 1A, the intensity of the EPR signal of the DEPMPO/OH adduct generated by an in vitro Fenton reaction was significantly higher in the control when compared to the strawberry extract, indicating a strong antiradical activity of the latter. Since the tissue of the strawberry fruit is a very complex system, several factors have a determinant role in different phenolics scavenging activity, such as the number and configuration of hydroxyl groups, glycosidic moieties, presence of both 2,3-double bond and a 4-oxo group, esterification process, etc. [50]. In such a complex medium, the chemistry behind the antioxidant activities of phenolic compounds is hard to define. Therefore, the spin-trapping method could provide useful information about the structure–antioxidant activity of phenolic compounds and their biological relevance in strawberry extracts.

DPPH is a free radical commonly used in EPR studies to measure the ability of plant-born anthocyanins to eliminate highly reactive species. The antioxidative potential of strawberry extracts is expressed as a decrease in DPPH radical concentration (Figure 1B). This is comparatively shown in Table 2, where the higher percentage indicates higher radical scavenging activity against DPPH. Thus, ‘Tea’, ‘Premy’, and ‘Joly’ were highly potent cultivars (over 70%), accompanied by ‘Laetitia’ (68.32%), while ‘Alba’, ‘Garda’, and ‘Arianna’ were cultivars with lower antiradical activity (below 50%).

### 2.3. Peroxidase (POD) Activity

**Figure 2:**
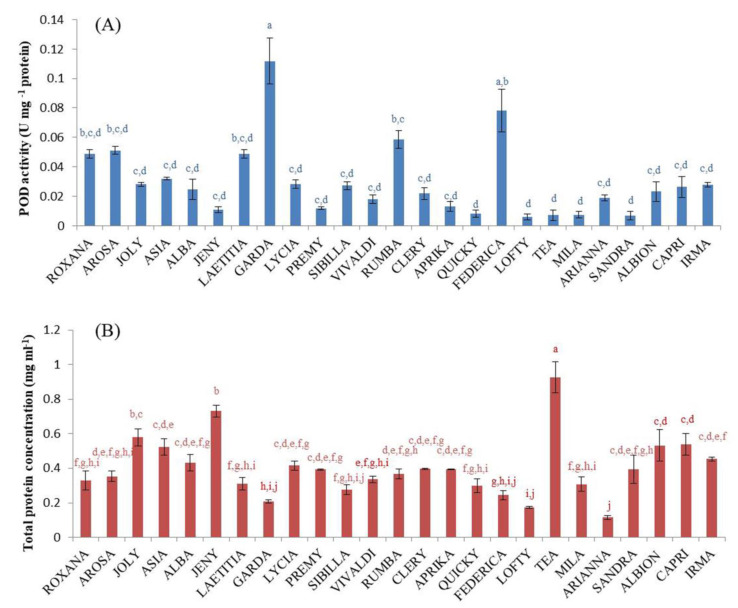
Specific activity of peroxidase (POD, U mg^−1^ prot, (**A**)) and total protein concentration (mg ml^−1^, (**B**)) in strawberry fruit extracts. Data are presented as means (*n* = 3) ± standard error (SE). Bars with different lowercase letters are significantly different (*p* < 0.05), as determined using the Duncan comparison test.

Plants have developed effective protection mechanisms against reactive oxygen species [14]. The antioxidant defense mechanisms include not only nonenzymic counterparts such as phenolics or ascorbic acid but also the enzymes such as peroxidases. The physiological role of peroxidase is attributed to phenolics oxidation including lignin biosynthesis [51]. Given that strawberry fruit is characterized by lignification processes, we examined specific POD activities in fruits of all tested cultivars (Figure 2A). Their specific activities were the highest in ‘Garda’, ‘Federica’, and ‘Rumba’ (0.11, 0.08, and 0.06 U mg^−1^ prot, respectively). The pattern of specific POD activity did not follow that of the total protein content (Figure 2B). Regardless of very high total protein concentrations, ‘Tea’, ‘Jeny’, and ‘Joly’ had low POD values (0.007, 0.01, and 0.03 U mg^−1^ prot, respectively). In the scarce literature data regarding enzyme activities in strawberry fruits, specific POD activities were presented in different units (U mg^−1^ FW, nkat g^−1^ FW), so they are not comparable with our results [25,52]. In addition, naturally occurring low POD activities in strawberry fruits are further compromised by the extraction procedure and manipulation during sample preparation; thus, in some studies, purification of the enzyme was carried out to gain maximal specific POD activities [53], which, therefore, significantly exceed our values. Specific POD activities in other raw fruit material were found to be higher in some species, such as raspberry (1–2 U mg^−1^ prot) [54] or significantly lower as in watermelon (0.003 U mg^−1^ prot) [55].

### 2.4. Polyphenols Profiles

One of the most characteristic classes of phenolic compounds that are widely distributed in the plant kingdom are flavonoids. They are subdivided into different subclasses depending on which carbon of the C ring the B ring is attached to and the saturation and oxidation status of the C ring (Figure S1). Flavonoids in which the B ring is in position 3 of the C ring are called isoflavones, while those in which the B ring is attached in position 2 can be further subdivided based on of the structural features of the C ring into several subgroups: flavones, flavonols, flavanones, flavanonols, flavanols or catechins, anthocyanins, and chalcones.

The basic flavonoid structure is aglycone, but they also occur as glycosides, and glycosidic linkage is usually located at positions 3 or 7 [56]. Occurrence, structure, position, and a total number of sugar moieties in flavonoids play an important role in the definition of their antioxidant activity. On the one hand, aglycones are more potent antioxidants than their corresponding glycosides, but on the other, the increasing degree of polymerization enhances the antioxidative effectiveness of procyanidins. Thus, procyanidin dimers and trimers are more effective antioxidants than monomeric flavonoids, while the activity of tetramers (i.e., oligomers) was found to be about four times higher than that of the preceding fractions [57]. 

Four phenolic compounds subgroups, anthocyanins, flavonols, flavan-3-ols, and hydroxycinnamic acids, which are considered as the main secondary metabolites in strawberry, were separated, identified, and quantified by HPLC in our study (Table 3, Table 4 and Table 5). 

The glycosylation pattern of tested cultivars showed that 92.23% of anthocyanins were presented as basic glycosides, while 7.77% appeared in acylated form (Table S1). A different pattern, with 44.94% of glycosides and 55.06% of acylated form, was perceived in derivatives of phenolics, while only 11.69% of quantified phenolic compounds were in a nonderivate, free form. 

Acquired flavonoid glycosides profiles of all tested cultivars indicated various sugar moieties: glucosides, acetylglucosides, rutinosides, malonylglucosides, glucuronides, and coumaroylglucoside. In total, 87.79% of anthocyanins were presented as glucosides, 7.34% as malonylglucosides, 4.44% as rutinosides, and only 0.43% as acetylglucosides. In derivatives of phenolics, the glycosylation pattern was different, where 53.50% of them occurred as coumaroylglucoside, 26.08% as glucuronides, 15.14% as glucosides, 3.71% as deoxyhexoside, and 1.56% as acetylglucosides.

Among five detected anthocyanin compounds, pelargonidin-3-*O*-glucoside (P-3-G) was predominant in all cultivars, making up almost 90% of the total anthocyanin content. The highest values were detected in ‘Joly’, ‘Asia’, ’Quicky’, and ‘Arianna’ (492.70, 487.08, 433.96, and 424.76 μg g^−1^ of FW, respectively). Similarly, in the study of Crecente-Campo et al. [58], P-3-G was the major anthocyanin contributor with the amount of 250–350 μg g^−1^ of FW, representing 91.5% of the total anthocyanins detected in strawberry fruit. 

**Figure 3:**
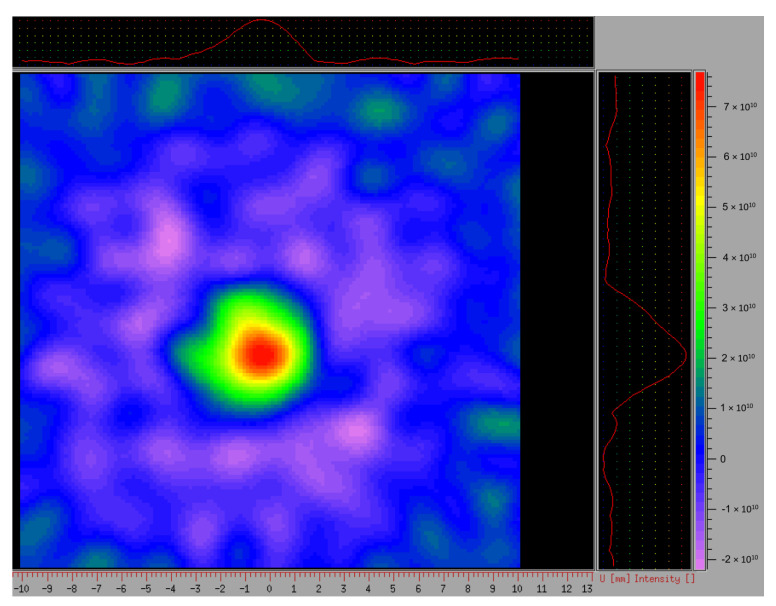
Visualization of the capacity of fresh fruit tissue to reduce spin-probe 3-carbamoyl proxyl (3CP). Two-dimensional EPR image of the Y-Z section.

Pelargonidin-3-*O*-malonylglucoside was the second most abundant anthocyanin in all cultivars, with an extremely high concentration in ‘Joly’ (156.58 µg eq P-3-G g^−1^ FW), while its concentration in all remaining cultivars was in the range from 1.60 to 54.89 µg eq P-3-G g^−1^ FW. Since the acylation of pelargonidin occurs mainly in achenes [59], it inspired us to investigate tissue distribution of the antioxidative potential of anthocyanins in fruit by 2D EPR imaging [60]. According to the color scale given in Figure 3, where red represents the lower and blue the higher spin-probe reduction values, the obtained gradient of free radicals along the fresh fruit cross-section confirmed that anthocyanins along with other antioxidants are mainly present at the fruit surface. Pelargonidin-3-*O*-rutinoside (P-3-R) and cyanidin-3-*O*-glucoside (C-3-G) have also been detected in strawberries with concentration ranges from 0.10 to 31.10 µg eq P-3-G g^−1^ FW and 1.03 to 9.50 µg g^−1^ FW, respectively, and their maximal values were recorded in ‘Laetitia’ and ‘Albion’ in the order already mentioned. Similar levels of P-3-R (13-55 µg g^−1^ FW) and C-3-G (9.8 µg g^−1^ FW) were reported for conventionally cultivated strawberries [58].

Flavonols are the most common and largest subgroup of flavonoids in fruits. They have a hydroxyl group in position 3 of the C ring (Figure S1), which may also be glycosylated. Derivatives of quercetin and kaempferol are the most abundant flavonols in strawberry, which was the case in our cultivars as well. A total of five flavonols were detected, of which only one compound was derived from quercetin and four from kaempferol (Table 5). Like the observations already reported [6], similar content of quercetin and myricetin was also detected in tested cultivars. Quercetin-3-glucuronide was dominant in ‘Tea’ and ‘Lofty’ (76.29 and 73.74 µg eq Querc g^−1^ FW), while in ‘Clery’ the lowest content was detected (2.59 µg eq Querc g^−1^ FW). In kaempferol compounds, different sugar moieties were represented: acetylglucosides, glucosides, glucuronides, and coumaroylglucosides. Cultivar ‘Lofty’ was predominant concerning kaempferol-3-acetylglucoside (20.38 µg eq Kaempf g^−1^ FW), followed by ‘Federica’, ‘Quicky’, ‘Tea’, and ‘Mila’ (15.40, 16.86, 13.86, and 10.29 µg eq Kaempf g^−1^ FW, respectively), while the remaining cultivars exhibited significantly lower values. Compared to other cultivars, ‘Mila’ was super-dominant in terms of kaempferol-3-glucoside, kaempferol-3-glucuronide, and kaempferol-3-coumaroylglucoside (127.09, 294.50, and 108.57 µg eq Kaempf g^−1^ FW, respectively), followed by ‘Arianna’ (83.58, 100.09, and 26.34 µg eq Kaempf g^−1^ FW, respectively) and ‘Tea’ (63.36, 81.31, and 98.94 µg eq Kaempf g^−1^ FW, respectively). The average mean values of most cultivars are in line with previously published content of kaempferol-3-glucoside (10–70 µg g^−1^ FW) and kaempferol-3-coumaroylglucoside (10–40 µg g^−1^ FW) in eastern strawberry [6].

Flavan-3-ols, represented by catechins, are compounds in which the hydroxyl group is always bound to position 3 of the C ring (Figure S1). Catechins seem to be the most powerful flavonoids for protection against reactive oxygen species radicals [14], also having good antibacterial, antiviral, and antifungal activity. Constitutive concentrations of catechins are changing during fruit development, being the highest in young (green) fruits to restrict fungal growth in this sensitive phase, thus assuming that flavanols’ biosynthesis is highly active in this developmental stage. A decrease in catechin concentration in the late ripening stage corresponds to a weak activity of the flavanol pathway in this stage [61].

In our study (Table 5), the highest contents of catechin were detected in ‘Asia’ and ‘Joly’ (76.73 and 75.92 µg g^−1^ FW), along with ‘Laetitia’ (66.56 µg g^−1^ FW). However, the HPLC profile of the most tested cultivars demonstrated lower values corresponding to those of 44.7 µg g^−1^ of the fresh edible weight of strawberry fruit reported in the literature [62].

Hydroxycinnamic acid derivatives are an important class of polyphenolic compounds originating from the Mevalonate–Shikimate biosynthesis pathways in plants, also serving as precursor molecules for flavonoids and anthocyanins [63]. A simple phenolic compound, such as *p*-coumaric acid, belongs to this important phenolic acid group. Previously, *p*-coumaric acid was identified as the major hydroxycinnamic acid in the ripe stage of four strawberry cultivars in the concentration range of 4.2–20.7 µg g^−1^ FW [64]. In our study, detected concentrations of *p*-coumaric acid were the highest in ‘Vivaldi’ and ‘Rumba’ cultivars with values of 11.37 µg g^−1^ FW, while in the remaining cultivars the concentrations were significantly lower (Table 4), which implies the possibility of the mild pre-fully ripe stage of our fruit. Coumaroyl glucose is an important derivative present in substantial quantities in strawberry fruit but with significant variation between tested cultivars. The highest values were found in ‘Asia’ (302.17 µg eq CA g^−1^ FW), followed by ‘Laetitia’ (252.72 µg eq CA g^−1^ FW), while almost 10 times smaller quantities were detected in ‘Federica’ (35.76 µg eq CA g^−1^ FW). Since the different hydroxycinnamic acids can be present in various derivatives among different cultivars [3], such discrepancy in content can be expected, when the apparent form (even being the dominant one) is selected.

Ellagic acid is a lactone-type, water-soluble phenolic compound mostly present in plant cell vacuoles in free and many various covalently bound forms [65]. The bound form predominates in most plants, but the free form is released during hydrolysis, which occurs in the human gastrointestinal tract under physiological conditions, thus enabling the utilization of plant-based ellagic acid to exert beneficial effects on human health [66]. The free form of ellagic acid and its derivative were detected in our cultivars in the concentration range of 1.27–34.23 µg g^−1^ FW, while the highest concentrations of both ellagic acid and ellagic acid deoxyhexoside were detected in ‘Laetitia’ (30.95 µg g^−1^ FW) and ‘Joly’ (34.23 µg eq EA g^−1^ FW), respectively (Table 4). The concentration of ellagic acid in fully ripe fruits of Turkish strawberry cultivars was found to be between 1.1 and 5.2 µg g^−1^ FW [64]. Since the decrease in the ellagic acid content between the green and fully ripe stages of strawberry fruit ranged from 2.8 to 8.5 times [65], its higher concentration in some of the tested cultivars confirms the possible mild pre-fully ripe stage of the fruit. The level of ellagic acid in strawberry fruits generally differs among cultivars, which is not only an effect of extraction and quantification methods but also the influence of growing conditions and practices.

From presented data, it is obvious that the majority of new cultivars are rich in bioactive phenolic compounds. Additional evaluation was to point out the most important group of analyzed compounds that stood out as a major contributor to antioxidant properties. As expected, the answer was not straightforward; the correlation of the sum of the compounds presented in each Table (Table 3, Table 4 and Table 5) with the estimated DPPH radical quenching ability of the apparent cultivar (Table 2) showed different patterns. After the analysis of ‘Alba’, ‘Garda’, ‘Joly’, and ‘Tea’ cultivars standing at the high/low margins of DPPH quenching activity, the highest correlation coefficient (r^2^ = 0.9413) was estimated for the sum of the flavonoids presented in Table 5, while considerably lower coefficients were calculated for the sum of anthocyanins (r^2^ = 0.2115) and phenolic acids (r^2^ = 0.0138). Thus, flavonoids seemed to be the major antioxidant foundation of strawberry fruit. However, when all of the analyzed cultivars were taken in to the account, no support for such a correlation was found among sums of all tested groups of phenolic compounds and DPPH quenching capacity. The right answer stands in the synergistic interaction of different amounts of different antioxidants present in each cultivar defining overall antioxidative potential. The aforementioned conclusion about the complexity of antioxidant interplay inside the fruit can never be grasped without comprehensive research of a number of various cultivars such as that which we presented.

## 3. Materials and Methods

### 3.1. Plant Materials

A total of 25 newly introduced strawberry cultivars, of which 22 are June-bearing cultivars (´Clery’, ‘Alba’, ‘Joly’, ‘Aprika’, ‘Asia’, ‘Arosa’, ‘Roxana’, ‘Jeny’, ‘Laetitia’, ‘Garda’, ‘Lycia’, ‘Premy’, ‘Sibilla’, ‘Quicky’, ‘Federica’, ‘Lofty’, ‘Tea’, ‘Mila’, ‘Arianna’, ‘Sandra’, ‘Vivaldi’, and ‘Rumba) and 3 are ever-bearing types (´Albion’, ‘Capri’, and ‘Irma´), were used in our experiment. All cultivars were grown in a plantation in Serbia located in the municipality of Šid (45°07′ N, 19°13′ E, 113 m a.s.l.). This region is characterized by a temperate continental climate, with a mean annual air temperature of 10.7 °C and a mean annual precipitation of 650 mm. The soil, a fine sandy loam, had a pH of 6.8 and medium to high levels of all nutrients. Cold-stored plants of tested strawberry cultivars were planted on raised double beds covered with black polyethylene foil in July 2020. Plant spacing was 30 cm × 30 cm. Drip irrigation with two laterals per raised bed and emitters at a 10 cm distance were applied. Fertigation was performed at a frequency in accordance with crop requirements previously reported by Tomić et al. [67].

Fully ripe fruit samples were collected in three repetitions per 20 fruits (60 fruits per cultivar) during the second harvest in the first year after planting (2021). Following the harvest, fruits were stored for a short time at −20 °C until chemical analysis to minimize the effect of postharvest factors.

### 3.2. Sample Preparation

Before chemical analysis, the whole fruits were carefully thawed, measured for exact weight, and homogenized using mortar and pestle. Phenolic compounds were extracted in 80% methanol at a ratio of 1:3 (*w/v*), while extraction of anthocyanins was performed in an extraction solution containing methanol/water/hydrochloric acid at a ratio of 70:30:5 by volume. After centrifugation at 13,000× *g* for 10 min at 4 °C, supernatants were used for further analyses.

Extracts for enzyme activity determination were prepared as follows: 1 g of homogenized fruit tissue were extracted in 2 mL 0.05 M sodium–phosphate buffer pH 7, containing 4% (*w/v*) polyvinylpyrrolidone (PVP) and 0.1% Triton X 100. After centrifugation at 13,000× *g* for 10 min at 4 °C, supernatants were used for spectrophotometric determination of peroxidase activities. Three extracts were prepared for each sample analyzed.

### 3.3. Spectrophotometric Measurements

Spectrophotometric determination of total phenolic content (TPC), total anthocyanin content (TACY), and protein concentration was performed on a Multiskan^®^ Spectrum UV/Vis spectrophotometer (Thermo Electron Corporation, Vantaa, Finland).

Determination of TPC in extracts was carried out with the use of the Folin−Ciocalteu spectrophotometric procedure using gallic acid (GA) as a standard for the calibration curve (0−340 μg of GA ml^−1^) [68]. Prepared standards and samples were mixed with 0.25 N Folin−Ciocalteu reagent and incubated for 3 min at 22 °C. Afterward, a 0.2 M sodium carbonate solution was added and incubated for 60 min at 22 °C. Absorbance was measured at 724 nm, and results were expressed as milligrams of gallic acid equivalent per gram of fresh weight (mg of GA equiv g^−1^ FW). 

A modified pH differential absorbance method was used to determine TACY [69]. For the analysis, two buffers were used: 0.025 M potassium chloride buffer at pH 1.0 and 0.4 M sodium acetate buffer at pH 4.5. The absorbance of strawberry extracts was read at 510 and 700 nm. Results were expressed as milligrams of pelargonidin-3-*O*-glucoside (ε = 17,330 l mol^−1^ cm^−1^) equivalents per 100 g of fresh weight (mg of P-3-G equiv 100 g^−1^ FW).

The Bradford method was used for the determination of protein concentration in samples after reading the absorbance at 595 nm, with bovine serum albumin (BSA) as a standard [70]. 

Spectrophotometric determination of peroxidases (POD) activity was performed on a Shimadzu spectrophotometer (UV-2501 PC 21, Kyoto, Japan). POD activity was measured by monitoring the formation of tetraguaiacol (e = 25.5 mM^−1^ cm^−1^) from guaiacol at 470 nm in the presence of H_2_O_2_ [71]. The reaction mixture consisted of 0.25% (*v*/*v*) guaiacol in 0.05 M sodium phosphate buffer pH 6.0 and 0.01 M H_2_O_2_. The total POD activity was divided by the protein concentration (in mg ml^−1^), and specific activity values were quoted as units per mg of proteins (U mg^−1^ prot).

### 3.4. Vitamin C

Vitamin C was quantified with the reflectometer set (Merck RQflex, Merck KGaA, Germany) as formerly described by Pantelidis et al. [72]. Results were expressed as mg ascorbic acid per 100 g of fresh weight (mg 100 g^−1^ FW).

### 3.5. HPLC Analysis

Analysis of individual phenolic compounds was performed by a reversed-phase HPLC-MS system consisted of 1525 binary pumps, thermostat, and 717+ autosampler connected to the Waters 2996 diode array and EMD 1000 Single quadrupole detector with ESI probe (Waters, Milford, MA, USA). Symmetry C-18 RP column (150 mm × 4.6 mm) packed with 5 µm particle diameter (Waters, Milford, MA, USA) connected to an appropriate guard column was used for separation. Binary gradient of mobile phases A (0.1% formic acid) and B (acetonitrile) were used at a flow of 1 ml per min, with the following gradient profile: in the first 20 min from 10 to 20% B; next 10 min of linear rise up to 40% B, followed by 15 min reverse to initial 10% B and additional 5 min of equilibration time. A postcolumn flow splitter (ASI, Richmond, CA, USA) with a 5/1 split ratio was used to obtain the optimal mobile phase inflow for the ESI probe. A DAD detector was set at 254, 315, and 510 nm for the detection of various classes of phenolic metabolites. For LC-MS analysis of anthocyanins, the positive ESI scan mode was used with the following parameters: capillary voltage 3.5 kV, cone voltage +35 V, and extractor and radio frequency (RF) lens voltages were 3.0 and 0.2 V, respectively. The negative ESI scan mode was used for other phenolic compounds with all parameters similar except for capillary and cone voltage: 3 kV and −30 V, respectively. When necessary for the quantification of metabolites, chromatograms were recorded in SIR mode. Source and desolvation temperatures were 120 °C and 360 °C respectively, with an N_2_ gas flow of 500 l\h. The data acquisition and spectral evaluation for peak confirmation were carried out by Waters Empower 2 Software (Waters, Milford, MA, USA).

### 3.6. Electron Paramagnetic Resonance (EPR) Spectroscopy 

To investigate the hydroxyl radicals (•OH) scavenging activity of strawberry extracts, EPR spectroscopy, in combination with the spin-trapping technique, was used following the procedure previously described [47,73]. In brief, the •OH-generating Fenton system consisted of 29 µL of the sample, which contained 25 µL of deionized water, 1 µL of strawberry extract (diluted 10 times), 2 µL of H_2_O_2_ (final concentration 0.35 mM), and 1 µL of spin trap DEPMPO (final concentration 3.5 mM). To initiate the reaction, 1 µL of FeSO_4_ (final concentration 0.15 mM) was added to the reaction solution that was immediately transferred into the gas-permeable Teflon tube and placed inside the resonator cavity. Recordings were made using the following experimental settings: center field 3500 G, microwave power 10 mW, microwave frequency 9.85 GHz, modulation frequency 100 kHz, modulation amplitude 1 G.

The same settings were used for recording EPR spectra of DPPH free radical degradation kinetics. The interaction of freshly prepared DPPH solution with strawberry-derived antioxidant compounds was studied by measuring the intensity of the DPPH EPR signal. Then, 28 µL of MeOH was mixed with 1 µL of 3.2 mM DPPH, and 1 µL of the strawberry extract was added before transferring the mixture into a 1 mm diameter Teflon tube and recording the EPR spectra [74]. In both experiments, solvent and sample blanks were provided for each assay to acquire control records.

For the 2D EPR imaging, strawberry fruit was incubated in the redox-sensitive, membrane-permeable aminoxyl spin-probe (10 mM 3-carbamoyl proxyl, 3CP), as described in Dragišić Maksimović et al. [60]. After 20 min of incubation, the whole fruit was placed inside the resonator cavity to visualize the in vivo arrangement of aminoxyl spin-probes in different fruit tissues. Endogenous ROS react with the nitroxide, reducing it to the EPR-silent hydroxylamine, thus diminishing the EPR signal. The spin-probe reduction rate is the measure of the redox status of the tissue. Signal intensity was color-coded in arbitrary EPRI units as specified by the color bar: yellow to red corresponds to high EPRI signal intensity, while green to blue indicates low to no EPRI signal intensity.

To perform L-band 2D EPR imaging measurements, the whole fruit was inserted into an ER540R36 resonator and measured using the following parameters: gradient strength 15 G cm^−1^, microwave power 10 mW, modulation frequency 30 kHz, modulation amplitude 1 G.

### 3.7. Statistical Analysis

Statistical analysis was performed using SPSS (IBM, Armonk, NY, USA). To examine significant differences in mean values of analyzed parameters between strawberry cultivars, analysis of variance (ANOVA) was used, while the Duncan test provided post hoc analysis. The level of statistical significance was set at 0.05.

## 4. Conclusions

In addition to yield, fruit chemical composition becomes very important as selection criteria toward health-promoting characteristics that can attribute to marketing prevalence as well as a consumer health benefit. This comparative study revealed that various strawberry cultivars differ in their phenolic profiles, so it is very important to select cultivars that are the most suitable for both commercial production and human health. 

Regardless of a large number of cultivars and monitored parameters that were included in this study, several cultivars stood out in terms of the phenolic profile together with other bioactive components: ‘Laetitia’, ‘Joly’, ‘Arianna’, ‘Tea’, and ‘Mila´. Intercultivar variations in phenolic profiles among them should be considered in breeding programs aimed at selecting promising cultivars with improved antioxidant capacity and nutraceutical properties. Increasing awareness of healthy nutrition in modern life places scientific studies of phytochemicals and their disease-preventing properties at the forefront, which is accompanied by higher consumer demands for ‘super fruits’. It opens up a new approach to personalized nutrition in relation to phenolic compounds naturally occurring in strawberries.

## Figures and Tables

**Table 1:** Total phenolic (TPC), anthocyanin (TACY), and vitamin C (Vit C) content in strawberry fruit extracts.

Cultivars	TPC (mg eq GA g^−1^ FW)	TACY (mg eq Pg-3-G 100 g^−1^ FW)	Vit C (mg 100 g^−1^ FW)
**Roxana**	0.49 ± 0.04 g,h	15.89 ± 0.23 g,h	52.80 ± 0.00 b
**Arosa**	0.71 ± 0.02 e,f,g,h	11.05 ± 0.16 h,i,j,k	55.17 ± 0.98 a,b
**Joly**	0.88 ± 0.09 b,c,d,e	22.14 ± 0.68 b,c,d,e,f	42.83 ± 1.27 d
**Asia**	0.66 ± 0.00 d,e,f,g,h	15.70 ± 1.62 g,h	42.83 ± 1.27 d
**Alba**	0.49 ± 0.03 h	7.90 ± 1.93 j,k,l	42.80 ± 1.04 d
**Jeny**	0.50 ± 0.12 f,g,h	3.08 ± 0.41 l	39.82 ± 0.42 d
**Laetitia**	1.07 ± 0.03 b,c	15.83 ± 1.22 f,g,h	56.32 ± 1.87 a
**Garda**	0.54 ± 0.03 f,g,h	6.79 ± 0.36 i,j,k,l	54.57 ± 1.53 a,b
**Lycia**	1.28 ± 0.08 a,b	8.23 ± 0.16 i,j,k,l	42.20 ± 0.85 d
**Premy**	1.53 ± 0.25 a	12.08 ± 2.48 h,i	34.17 ± 1.65 e,f
**Sibilla**	1.33 ± 0.15 a	8.66 ± 0.64 i,j,k	32.27 ± 0.49 f
**Vivaldi**	1.37 ± 0.03 a	24.92 ± 1.91 a,b,c	46.70 ± 0.92 c
**Rumba**	1.40 ± 0.10 a	19.46 ± 3.25 d,e,f,g	49.07 ± 1.20 c
**Clery**	0.81 ± 0.05 c,d,e,f	8.88 ± 0.51 i,j,k	46.33 ± 1.27 c
**Aprika**	1.41 ± 0.10 a	11.44 ± 0.26 h,i,j	34.90 ± 0.52 e,f
**Quicky**	1.15 ± 0.06 b	22.56 ± 0.60 b,c,d	36.27 ± 0.67 e
**Federica**	0.89 ± 0.11 b,c,d,e	18.95 ± 0.78 c,d,e,f	35.80 ± 0.52 e,f
**Lofty**	0.93 ± 0.08 b,c,d	23.81 ± 0.50 b,c,d	33.93 ± 1.10 e,f
**Tea**	0.76 ± 0.02 d,e,f,g	18.47 ± 0.69 e,f,g	35.20 ± 0.00 e,f
**Mila**	0.77 ± 0.04 d,e,f,g	20.97 ± 0.20 c,d,e	34.60 ± 1.04 e,f
**Arianna**	0.90 ± 0.03 b,c,d,e	26.05 ± 0.53 a,b	35.80 ± 0.52 e,f
**Sandra**	0.87 ± 0.03 b,c,d,e	30.60 ± 0.15 a	36.57 ± 0.75 e
**Albion**	0.71 ± 0.01 e,f,g,h	11.18 ± 1.00 h,i,j	48.10 ± 2.08 c
**Capri**	0.69 ± 0.05 d,e,f,g,h	5.86 ± 0.26 k,l	41.04 ± 2.01 d
**Irma**	0.46 ± 0.01 h	9.11 ± 0.41 i,j,k	42.23 ± 1.20 d

Data are presented as means (*n* = 3) ± standard error (SE). Values within a column with different lowercase letters are significantly different (*p* < 0.05), as determined using the Duncan comparison test. FW, fresh weight; GA, gallic acid; Pg-3-G, pelargonidin-3-*O*-glucoside.

**Table 2:** •OH and DPPH radical scavenging activity (%) of strawberry fruit extracts estimated by electron paramagnetic resonance (EPR) spectroscopy in combination with the spin-trapping technique.

Cultivars	•OH	DPPH
**Roxana**	93.85	54.16
**Arosa**	95.53	61.42
**Joly**	94.71	70.36
**Asia**	94.78	62.75
**Alba**	94.71	46.39
**Jeny**	94.90	61.20
**Laetitia**	94.82	68.32
**Garda**	95.31	47.71
**Lycia**	94.71	56.98
**Premy**	94.04	71.61
**Sibilla**	94.63	61.56
**Vivaldi**	95.27	65.11
**Rumba**	94.23	62.89
**Clery**	94.37	68.13
**Aprika**	93.63	63.95
**Quicky**	93.74	62.89
**Federica**	93.67	59.71
**Lofty**	94.67	66.79
**Tea**	94.97	78.88
**Mila**	94.34	53.00
**Arianna**	93.52	48.60
**Sandra**	94.04	63.98
**Albion**	94.71	59.06
**Capri**	94.49	58.63
**Irma**	94.00	58.77

**Table 3:** Content (μg g^−1^ of FW) of anthocyanins in strawberry fruit extracts.

Cultivars	Pelargonidin-3-*O*-glucoside	Pelargonidin-3-*O*-acetylglucoside (P-3-G Equiv.)	Pelargonidin-3-*O*-rutinoside (P-3-G Equiv.)	Pelargonidin-3-*O*-malonylglucoside (P-3-G Equiv.)	Cyanidin-3-*O*-glucoside
**Roxana**	264.27 ± 21.64 f,g,h	5.20 ± 0.82 b	23.80 ± 2.12 a,b	1.90 ± 0.85 i,j	3.20 ± 0.57 b,c,d,e,f,g,h
**Arosa**	230.88 ± 36.62 d,e,f,g	3.03 ± 0.00 c	20.35 ± 9.83 c,d,e,f	39.15 ± 0.21 c,d,e	1.60 ± 0.28 h
**Joly**	492.70 ± 12.09 a,b	n.d.	17.60 ± 0.28 c,d,e	156.58 ± 3.82 a	2.55 ± 0.21 e,f,g,h
**Asia**	487.08 ± 24.33 a	8.30 ±0.66 a	22.33 ± 1.63 b,c	2.10 ± 0.21 i,j	1.45 ± 0.21 h
**Alba**	218.78 ± 8.63 h,i,j	0.67 ± 0.14 e	12.63 ± 1.81 e,f,g,h	39.40 ± 1.27 b,c	3.95 ± 0.64 b,c,d,e,f,g,h
**Jeny**	81.14 ± 7.57 k	n.d.	0.10 ± 0.00 l	22.95 ± 0.21 e,f,g	2.65 ± 0.92 e,f,g,h
**Laetitia**	357.81 ± 27.65 b,c,d,e,f	0.33 ± 0.06 e	31.10 ± 1.98 a	37.50 ± 0.21 b,c,d	3.20 ± 0.28 d,e,f,g,h
**Garda**	191.63 ± 14.07 j	0.27 ± 0.00 e	22.80 ± 0.14 b,c,d	44.80 ± 3.18 b	4.67 ± 0.67 b,c,d,e,f
**Lycia**	206.35 ± 17.23 i,j	0.40 ± 0.07 e	10.69 ± 0.21 e,f,g,h	38.90 ± 2.76 b,c	5.42 ± 1.19 b,c,d,e,f,g
**Premy**	190.57 ± 8.51 i,j	n.d.	13.07 ± 0.40 e,f,g,h	12.27 ± 0.49 f,g,h,i,j	5.60 ± 0.20 b,c
**Sibilla**	171.44 ± 7.13 j	0.27 ± 0.06 e	11.29 ± 0.98 e,f,g,h,i	12.20 ± 0.14 f,g,h,i,j	2.52 ± 0.79 f,g,h
**Vivaldi**	301.44 ± 10.89 e,f,g,h	0.50 ± 0.07 e	10.83 ± 1.19 e,f,g,h,i,j	24.53 ± 0.85 d,e,f	4.95 ± 0.79 b,c,d,e
**Rumba**	222.20 ± 36.43 h,i,j	0.13 ± 0.06 e	12.51 ± 0.79 e,f,g,h	34.87 ± 3.75 b,c,d	1.03 ± 0.00 h
**Clery**	42.00 ± 6.25 l	n.d.	3.36 ± 0.59 k	3.00 ± 0.49 h,i,j	6.16 ± 0.40 b
**Aprika**	182.36 ± 1.98 i,j	n.d.	15.12 ± 2.91 c,d,e,f,g	12.37 ± 0.35 f,g,h,i,j	6.58 ± 1.78 b
**Quicky**	433.96 ± 8.91 a,b,c	2.40 ± 0.42 c,d	12.60 ± 2.12 e,f,g,h	20.40 ± 1.30 e,f,g	3.10 ± 0.21 c,d,e,f,g,h
**Federica**	343.77 ± 34.15 c,d,e,f,g	6.10 ± 0.21 b	5.25 ± 1.91 j,k,l	2.40 ± 0.85 i,j	10.60 ± 1.91 a
**Lofty**	301.47 ± 8.91 e,f,g,h	n.d.	17.40 ± 1.27 c,d,e,f	12.20 ± 1.13 f,g,h,i,j	1.20 ± 0.21 h
**Tea**	276.97 ± 3.82 g,h,i	3.00 ± 0.42 c	19.00 ± 1.91 c,d,e	n.d.	3.40 ± 0.46 b,c,d,e,f,g,h
**Mila**	334.77 ± 1.48 d,e,f,g	n.d.	27.40 ± 0.85 a,b	11.90 ± 0.28 f,g,h,i,j	4.30 ± 0.46 b,c,d,e,f,g
**Arianna**	424.76 ± 8.10 a,b,c,d	0.70 ± 0.00 e	8.25 ± 2.12 h,i,j,k,l	15.17 ± 0.67 e,f,g,h,i	2.40 ± 0.85 g,h
**Sandra**	395.36 ± 7.65 b,c,d,e	n.d.	4.50 ± 0.79 i,j,k,l	16.96 ± 0.42 e,f,g,h	5.90 ± 0.42 b,c
**Albion**	257.62 ± 54.65 e,f,g,h	0.90 ± 0.35 e	23.20 ± 7.07 c,d,e,f	54.89 ± 15.70 b	9.50 ± 0.99 a
**Capri**	199.03 ± 5.73 h,i,j	1.80 ± 0.14 c,d	12.50 ± 0.57 d,e,f,g,h	7.20 ± 1.70 g,h,i,j	3.80 ± 0.17 b,c,d,e,f,g,h
**Irma**	202.43 ± 4.60 h,i,j	1.75 ± 0.07 c,d	9.75 ± 0.92 g,h,i,j,k	1.60 ± 0.42 i,j	4.20 ± 0.28 b,c,d,e

Data are presented as means (*n* = 3) ± standard error (SE). Values within a column with different lowercase letters are significantly different (*p* < 0.05), as determined using the Duncan comparison test. Labels in parentheses are analogous with micrograms of corresponding anthocyanin equivalents per gram of fresh weight (FW). P-3-G equiv., pelargonidin-3-glucoside equivalents; n.d., not detected.

**Table 4:** Content (μg g^−1^ of FW) of phenolic acids and their derivatives in strawberry fruit extracts.

Cultivars	Ellagic Acid	Ellagic Acid Deoxyhexoside (EA Equiv.)	*p*-Coumaric Acid	*p*-Coumaroyl Glucose (CA Equiv.)
**Roxana**	18.60 ± 0.71 c,d,e,f,g	15.18 ± 0.85 e,f	1.87 ± 0.40 h,i,j	122.37 ± 11.54 d,e,f
**Arosa**	13.90 ± 6.08 b,c,d,e,f	21.35 ± 3.04 b,c,d	2.87 ± 0.51 f,g,h	96.23 ± 9.42 f,g
**Joly**	33.40 ± 4.81 b,c,d	34.23 ± 4.31 a	1.55 ± 0.07 i,j	182.97 ± 15.77 c
**Asia**	30.60 ± 1.61 a,b,c	21.51 ± 0.42 b,c,d	4.27 ± 0.40 e,f	302.17 ± 1.65 a
**Alba**	13.33 ± 0.25 e,f,g	7.29 ± 0.26 g,h	4.25 ± 0.21 e,f	135.35 ± 3.06 c,d,e
**Jeny**	18.15 ± 2.33 b,c,d,e,f	16.95 ± 0.14 d,e	4.10 ± 0.71 e,f	112.54 ± 20.72 e,f
**Laetitia**	30.95 ± 6.72 a	23.58 ± 0.92 b	3.80 ± 0.71 f,g	252.72 ± 28.25 b
**Garda**	7.90 ± 0.44 g,h	9.39 ± 0.42 g	2.05 ± 0.21 h,i,j	164.49 ± 3.77 c
**Lycia**	13.73 ± 0.00 c,d,e,f,g	17.13 ± 0.49 c,d,e	3.53 ± 0.64 f,g,h	135.35 ± 14.83 c,d,e
**Premy**	10.58 ± 0.59 f,g	6.86 ± 1.55 g,h	6.57 ± 0.93 c,d,e	67.59 ± 0.28 h,i,j
**Sibilla**	10.61 ± 0.72 f,g	3.40 ± 0.95 h	6.17 ± 0.67 d,e	65.94 ± 1.91 h,i,j
**Vivaldi**	12.63 ± 0.59 e,f,g	2.83 ± 0.55 h	11.37 ± 0.78 a	111.72 ± 2.83 e,f
**Rumba**	14.48 ± 1.53 e,f,g	2.96 ± 0.81 h	11.37 ± 1.20 a	63.33 ± 3.69 h,i,j
**Clery**	31.23 ± 3.14 a,b	3.20 ± 0.52 h	1.20 ± 0.14 j	75.03 ± 3.32 g,h,i
**Aprika**	11.95 ± 1.18 e,f,g	6.76 ± 1.34 g,h	6.60 ± 0.07 c,d,e	65.94 ± 2.76 h,i,j
**Quicky**	14.45 ± 0.78 c,d,e,f,g	3.00 ± 0.80 h	8.53 ± 0.35 b	55.79 ± 6.79 j,k
**Federica**	5.55 ± 0.64 h	1.70 ± 0.61 h	7.50 ± 0.71 c,d	35.76 ± 4.21 l
**Lofty**	21.10 ± 2.12 d,e,f,g	2.13 ± 0.25 h	5.83 ± 0.23 d,e	41.96 ± 2.80 k,l
**Tea**	11.55 ± 0.07 e,f,g	1.27 ± 0.15 h	5.73 ± 0.40 d,e	86.79 ± 1.41 g,h
**Mila**	9.60 ± 1.48 g	1.83 ± 0.06 h	6.43 ± 0.21 c,d,e	73.04 ± 1.34 g,h,i
**Arianna**	9.90 ± 0.49 f,g	2.13 ± 0.29 h	8.73 ± 0.38 b	74.34 ± 1.06 g,h,i
**Sandra**	14.70 ± 2.12 e,f,g	1.73 ± 0.06 h	8.10 ± 0.14 b,c	61.34 ± 0.92 i,j,k
**Albion**	18.15 ± 0.07 c,d,e,f,g	10.72 ± 0.21 f,g	1.23 ± 0.29 j	73.92 ± 10.36 g,h,i
**Capri**	22.80 ± 3.11 b,c,d,e	22.84 ± 0.07 b,c	6.00 ± 0.85 d,e	171.48 ± 11.30 c
**Irma**	9.55 ± 2.76 f,g	9.66 ± 1.06 g	2.93 ± 0.06 f,g,h	220.92 ± 20.48 b

Data are presented as means (*n* = 3) ± standard error (SE). Values within a column with different lowercase letters are significantly different (*p* < 0.05), as determined using the Duncan comparison test. Labels in parentheses are analogous with micrograms of corresponding anthocyanin equivalents per gram of fresh weight (FW). EA equiv., ellagic acid equivalents; CA equiv., *p*-coumaric acid equivalents.

**Table 5:** Content (μg g^−1^ of FW) of flavonols and flavan-3-ols in strawberry fruit extracts.

Cultivars	Catechin	Quercetin-3-glucuronide (Querc Equiv.)	Kaempferol-3-acetylglucoside (Kaempf Equiv.)	Kaempferol-3-glucoside (Kaempf Equiv.)	Kaempferol-3-glucuronide (Kaempf Equiv.)	Kaempferol-3-coumaroyglucoside (Kaempf Equiv.)
**Roxana**	43.56 ± 3.82 d,e,f,g	5.95 ± 0.21 i,j	1.15 ± 0.07 f,g	6.34 ± 0.56 l	3.32 ± 0.20 h	20.83 ± 4.42 e,f
**Arosa**	35.69 ± 3.85 g,h,i,j	15.30 ± 3.68 g	1.20 ± 0.17 f,g	5.94 ± 1.56 l	18.26 ± 1.93 f	10.61 ± 0.28 g,h
**Joly**	75.92 ± 1.62 a	11.60 ± 1.31 h,i	1.80 ± 0.14 e,f	42.08 ± 0.25 e,f	77.97 ± 4.98 c	30.56 ± 1.32 d
**Asia**	76.73 ± 3.78 a	8.77 ± 0.47 h,i	1.67 ± 1.07 e,f	16.09 ± 0.49 h,i	3.66 ± 0.40 h,i	9.33 ± 0.23 h
**Alba**	52.27 ± 6.35 c,d	11.05 ± 1.06 h	1.40 ± 0.30 f,g	13.14 ± 0.10 i,j	9.59 ± 0.09 g	14.51 ± 2.70 g
**Jeny**	22.10 ± 0.84 j	40.25 ± 1.77 d	1.25 ± 0.07 f,g	20.50 ± 3.27 g,h	14.53 ± 0.71 f,g	21.48 ± 6.61 e,f
**Laetitia**	66.56 ± 2.96 b	23.50 ± 1.98 f	1.57 ± 0.15 e,f	28.58 ± 0.13 g	15.83 ± 0.83 f,g	20.97 ± 5.65 e,f
**Garda**	27.44 ± 1.34 i,j	11.95 ± 1.48 h	1.80 ± 0.42 e,f	3.95 ± 0.21 l	8.22 ± 0.62 g,h	9.76 ± 2.23 h
**Lycia**	30.92 ± 1.59 h,i,j,k	19.81 ± 2.37 f,g	0.94 ± 0.07 g,h	19.76 ± 2.85 g,h	7.76 ± 0.56 g	48.03 ± 2.39 c
**Premy**	29.77 ± 0.76 h,i,j	19.49 ± 2.96 f,g	2.44 ± 0.28 e	16.23 ± 2.88 h,i	73.05 ± 0.95 c	3.22 ± 0.22 i
**Sibilla**	27.31 ± 1.53 i,j	20.39 ± 1.80 f,g	1.46 ± 0.49 f,g	17.86 ± 0.21 h,i	44.00 ± 2.88 e	3.49 ± 0.20 i
**Vivaldi**	50.72 ± 1.83 c,d	6.12 ± 2.04 i,j	1.36 ± 0.12 f,g	19.22 ± 0.76 g,h	67.81 ± 3.01 c,d	3.92 ± 0.65 i
**Rumba**	36.60 ± 4.37 g,h,i,j	8.02 ± 1.02 h	0.80 ± 0.11 h	51.30 ± 5.25 e	72.56 ± 3.76 c	4.41 ± 0.22 i
**Clery**	6.95 ± 0.22 k	2.59 ± 0.49 j	1.70 ± 0.40 e,f,g	47.51 ± 1.52 e,f	48.28 ± 2.10 d,e	3.42 ± 0.24 i
**Aprika**	28.98 ± 0.44 i,j	15.90 ± 0.54 h	0.94 ± 0.08 g,h	12.95 ± 1.16 i,j	64.78 ± 2.02 c	4.12 ± 0.65 i
**Quicky**	54.89 ± 3.96 c	19.33 ± 0.84 f,g	16.86 ± 5.21 b	77.86 ± 0.39 c,d	20.05 ± 3.37 f	32.94 ± 9.10 d
**Federica**	43.33 ± 3.24 d,e,f,g	31.36 ± 5.51 e	15.40 ± 2.13 b	80.12 ± 17.55 b,c	37.14 ± 0.10 e	99.31 ± 7.37 b
**Lofty**	37.60 ± 0.44 g,h,i,j	73.74 ± 0.64 b	20.38 ± 1.52 a	48.09 ± 1.61 e,f	60.16 ± 4.66 c,d	27.10 ± 9.34 d,e
**Tea**	48.11 ± 1.51 c,d.e	76.29 ± 0.28 a	13.86 ± 2.16 b,c	63.36 ± 16.45 d,e	81.31 ± 3.13 c	98.94 ± 17.75 b
**Mila**	39.61 ± 1.07 f,g,h,i	50.73 ± 4.60 c	10.29 ± 2.01 c,d	127.09 ± 27.73 a	294.50 ± 5.39 a	108.57 ± 51.59 a
**Arianna**	51.89 ± 0.31 c,d	29.66 ± 0.07 e,f	6.15 ± 0.27 d	83.58 ± 2.48 b,c	100.09 ± 14.54 b	26.34 ± 12.21 d,e
**Sandra**	31.33 ± 1.27 h,i,j,k	34.00 ±1.06 e	7.92 ± 0.50 d	79.56 ± 37.99 b,c	9.07 ± 1.47 g	41.26 ± 13.03 c
**Albion**	32.73 ± 5.85 h,i,j	18.90 ± 0.92 f,g	1.35 ± 0.07 f,g	20.18 ± 1.94 g,h	14.07 ± 3.46 f,g	30.05 ± 6.22 d
**Capri**	41.23 ± 2.41 e,f,g,h	33.50 ± 0.42 e,f	6.44 ± 3.04 d	51.58 ± 0.47 e	1.62 ± 0.43 j	11.88 ± 2.37 g,h
**Irma**	37.28 ± 1.17 g,h,i,j	11.95 ± 0.78 h,i	1.65 ± 0.07 e,f	11.14 ± 0.58 j	5.36 ± 0.29 h	37.65 ± 5.09 c,d

Data are presented as means (*n* = 3) ± standard error (SE). Values within a column with different lowercase letters are significantly different (*p* < 0.05), as determined using the Duncan comparison test. Labels in parentheses are analogous with micrograms of corresponding phenolic equivalents per gram of fresh weight (FW). Querc equiv., quercetin equivalents; Kaempf equiv., kaempferol equivalents.

## Data Availability

All new research data were presented in this contribution.

## Supplementary Materials

The following supporting information can be downloaded at: https://www.mdpi.com/article/10.3390/plants11243566/s1. Table S1: The glycosylation pattern (%) of phenolic compounds in strawberry fruit extracts. Figure S1: Basic structure of flavonoids.
