# Supplementary material for: Rich in Phenolics—Strong Antioxidant Fruit? Comparative Study of 25 Strawberry Cultivars
Source: Plants (Basel). 2022 Dec 17;11(24):3566. doi: 10.3390/plants11243566 (PMC9784063; doi:10.3390/plants11243566)
Supplement: Supplementary file 1 [file plants-11-03566-s001.zip › plants-2087203-supplementary.pdf]

plants

MDPI

Article

Rich in Phenolics—Strong Antioxidant Fruit? Comparative Study of 25 Strawberry Cultivars

Dragica M. Milosavljević [1], Vuk M. Maksimović [1], Jasminka M. Milivojević [2], Đura J. Nakarada [3], Miloš D. Mojović [3] and Jelena J. Dragišić Maksimović [1,*]

Table S1. The glycosylation pattern (%) of phenolic compounds in strawberry fruit extracts.

**Anthocyanins**	Glycosides	92.23
	Acetyl form	7.77
	Glucoside	87.79
	Rutinoside	4.44
	Acetylglucoside	0.43
	Malonylglucoside	7.34
**Derivatives of phenols**	Glycosides	44.94
	Acetyl form	55.06
	Glucuronide	26.08
	Glucoside	15.14
	Acetylglucoside	1.56
	Coumaroyl glucoside	53.50
	Deoxyhexoside	3.71
**Free forms**	11.69	

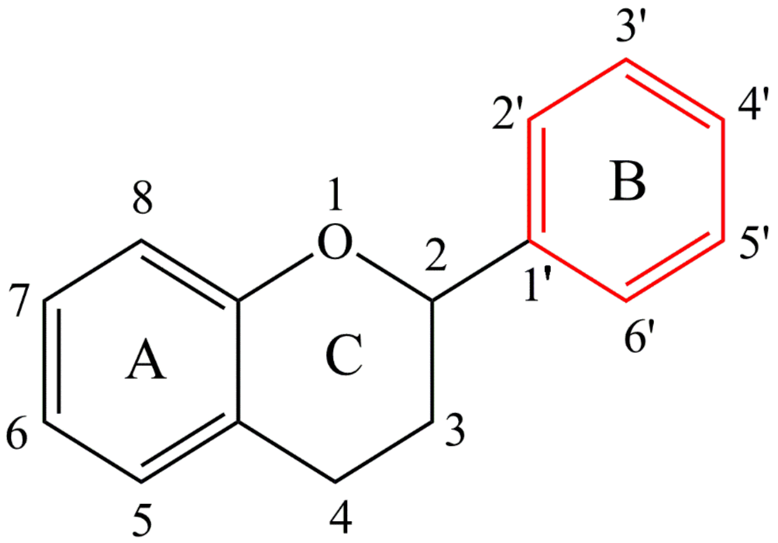

Figure S1. Basic structure of flavonoids.
